# Supplementary material for: Handheld Ultrasound Devices Used by Newly Certified Operators for Pneumonia in the Emergency Department—A Diagnostic Accuracy Study
Source: Diagnostics (Basel). 2024 Aug 30;14(17):1921. doi: 10.3390/diagnostics14171921 (PMC11394211; doi:10.3390/diagnostics14171921)
Supplement: Supplementary file 1 [file diagnostics-14-01921-s001.zip › Files S5 and S6 - Tabulation of agreement.pdf]

File S5 – Tabulation of FLUS agreement between operators and expert

	FLUS expert: Not Pneumonia	FLUS expert: Pneumonia	Total
FLUS operator : Not pneumonia	8	3	11
FLUS operator: Pneumonia	4	2	6
Total	12	5	17

1 missing FLUS Scanning due to damage of saved video clip. FLUS= Focused lung ultrasound.

File S6 – Tabulation of FLUS interobserver agreement

	FLUS operator A: Not Pneumonia	FLUS operator A: Pneumonia	Total
FLUS operator B : Not pneumonia	4	2	6
FLUS operator B: Pneumonia	2	1	3
Total	6	3	9

FLUS = Focused lung ultrasound.
