# Supplementary material for: Handheld Ultrasound Devices Used by Newly Certified Operators for Pneumonia in the Emergency Department—A Diagnostic Accuracy Study
Source: Diagnostics (Basel). 2024 Aug 30;14(17):1921. doi: 10.3390/diagnostics14171921 (PMC11394211; doi:10.3390/diagnostics14171921)
Supplement: Supplementary file 1 [file diagnostics-14-01921-s001.zip › File S1 - Focused Lung Ultrasound Assessment Template.pdf]

File S1: Focused Lung Ultrasound Assessment Template

FLUS

Record ID ______________________

LUS Logistics

	Sitting	Supine (lying on back)	Prone (lying on stomach)	Left lateral decubitus (lying on left side)	Right lateral decubitus (lying on right side)
Patients position	☐	☐	☐	☐	☐

Have all FLUS zones been scanned?

- ◯ No
- ◯ Yes

Which zones could not be scanned?

- ☐ 1R
- ☐ 2R
- ☐ 3R
- ☐ 4R
- ☐ 5R
- ☐ 6R
- ☐ 7R
- ☐ 1L
- ☐ 2L
- ☐ 3L
- ☐ 4L
- ☐ 5L
- ☐ 6L
- ☐ 7L

Start scan ______________________

End scan ______________________

Total time ______________________

Lung sliding

Lung sliding in all zones?

- ◯ No
- ◯ Yes

Missing in which zone(s)?	☐ 1R<br>☐ 2R<br>☐ 3R<br>☐ 4R<br>☐ 5R<br>☐ 6R<br>☐ 7R<br>☐ 1L<br>☐ 2L<br>☐ 3L<br>☐ 4L<br>☐ 5L<br>☐ 6L<br>☐ 7L
Lunge puls	○ No<br>○ Yes
B-lines (in zones WITHOUT lung sliding)	○ No<br>○ Yes
Lung point	○ No<br>○ Yes
Lung point located in which zones?	☐ 1R<br>☐ 2R<br>☐ 3R<br>☐ 4R<br>☐ 5R<br>☐ 6R<br>☐ 7R<br>☐ 1L<br>☐ 2L<br>☐ 3L

AFLUS: Focused lung ultrasound
