# Supplementary material for: Handheld Ultrasound Devices Used by Newly Certified Operators for Pneumonia in the Emergency Department—A Diagnostic Accuracy Study
Source: Diagnostics (Basel). 2024 Aug 30;14(17):1921. doi: 10.3390/diagnostics14171921 (PMC11394211; doi:10.3390/diagnostics14171921)
Supplement: Supplementary file 1 [file diagnostics-14-01921-s001.zip › File S2 - Template for Radiological Assessments of CXR.pdf]

File S2: Template for Radiological Assessments of CXR

CXR

Record ID	______________________
Assessment of CXR - Start	______________________
Standing or supine X-ray:	◯ Standing X-ray<br>◯ Supine X-ray
Is the entire thorax visible on the chest X-ray?	◯ No<br>◯ Yes
Radiological findings on chest X-ray:	☐ No abnormalities<br>☐ Infiltrate<br>☐ Pleural effusion<br>☐ Pulmonary congestion/pulmonary edema/accentuated lung markings<br>☐ Pneumothorax<br>☐ Changes suspicious for malignancy<br>☐ Other
Location of infiltrate:	☐ Right upper lobe<br>☐ Right middle lobe<br>☐ Right lower lobe<br>☐ Left upper lobe<br>☐ Left lower lobe<br>((Register all affected))
Location of pleural effusion	☐ Right upper lobe<br>☐ Right middle lobe<br>☐ Right lower lobe<br>☐ Left upper lobe<br>☐ Left lower lobe<br>((Register all affected lobes))
Location of pulmonary congestion	☐ Right upper lobe<br>☐ Right middle lobe<br>☐ Right lower lobe<br>☐ Left upper lobe<br>☐ Left lower lobe<br>((Register all affected lobes))
Location of pneumothorax	☐ Right upper lobe<br>☐ Right middle lobe<br>☐ Right lower lobe<br>☐ Left upper lobe<br>☐ Left lower lobe<br>((Register all affected lobes))
Location of lesion suspicious for malignancy	☐ Right upper lobe<br>☐ Right middle lobe<br>☐ Right lower lobe<br>☐ Left upper lobe

Describe other findings	__________________________________
Location of other findings on chest X-ray	☐ Right upper lobe<br>☐ Right middle lobe<br>☐ Right lower lobe<br>☐ Left upper lobe<br>☐ Left lower lobe<br>((Register all affected lobes))
Is pneumonia the most likely diagnosis?	○ no<br>○ yes
Suspected diagnosis based on chest x-ray	☐ Normal findings<br>☐ Pneumonia<br>☐ Non-cardiogenic pulmonary oedema<br>☐ Cardiogenic pulmonary oedema<br>☐ Pneumonia with parapneumoic effusion<br>☐ Empyema<br>☐ Pleural effusion of unknown origin<br>☐ Pulmonary embolism<br>☐ Pneumothorax<br>☐ COPD<br>☐ Asthma<br>☐ Interstitial lung disease<br>☐ Malignancy<br>☐ Other<br>((Several diagnoses may be chosen))
Describe other suspected diagnosis	__________________________________<br>((Several diagnoses may be chosen))
Description of chest X-ray - End	__________________________________

CXR: chest x-ray
