# Supplementary material for: Handheld Ultrasound Devices Used by Newly Certified Operators for Pneumonia in the Emergency Department—A Diagnostic Accuracy Study
Source: Diagnostics (Basel). 2024 Aug 30;14(17):1921. doi: 10.3390/diagnostics14171921 (PMC11394211; doi:10.3390/diagnostics14171921)
Supplement: Supplementary file 1 [file diagnostics-14-01921-s001.zip › File S3 -Template for Radiological Assessments of CT.pdf]

File S3: Template for Radiological Assessments of CT

Translation: ja=Yes; nej=no;

CT

Record ID	________________
Patienten er ikke inkluderet i pneumoni-sporet eller er ikke blevet henvist til CT.<br><br>Marker instrumentet som complete	
Patientens CPR: [basic_cpr]<br>Indlagt: [admission_time]	
Time for CT assessment begins	________________
Entire chest cavity present on CT images	○ Ja<br>○ Nej

Heart, mediastinum and central vessels

Pericardial or heart pathology	○ Ja<br>○ Nej
Describe pericardial or heart pathology	________________
Mediastinal pathology	○ Ja<br>○ Nej
Type of mediastinal pathology	○ Lymphadenopathy<br>○ Other
Describe other mediastinal pathology	________________
Dilated truncus pulmonalis	○ Ja<br>○ Nej
Other pathology in central vessels	○ Ja<br>○ Nej<br>((E.g. aorta, v cava))
Describe possible pathology in central vessels	________________

Hila and lung vessels

Pathology in lung hila	◯ Ja<br>◯ Nej
Placement of pathology in lung hila	◯ Right<br>◯ Left<br>◯ Bilateral
Type of hilar pathology	◯ Lymphadenopathy<br>◯ Other
Describe other hilar pathology	__________________________________
Dilated pulmonary arteries	◯ Ja<br>◯ Nej

Lung parenchyma

Consolidation present	◯ Ja<br>◯ Nej
Consolidation pattern	☐ Pneumonia pattern<br>☐ Atelectasis pattern<br>☐ Nodule (< 31 mm)<br>☐ Mass (> 30 mm)<br>☐ Other<br>((Register all types of opacities identified))
Placement of pneumonic consolidation	☐ Right upper lobe<br>☐ Right middle lobe<br>☐ Right lower lobe<br>☐ Left upper lobe<br>☐ Left lower lobe<br>((Register all affected))
Placement of consolidation due to atelectasis	☐ Right upper lobe<br>☐ Right middle lobe<br>☐ Right lower lobe<br>☐ Left upper lobe<br>☐ Left lower lobe<br>((Register all affected lobes))
Placement of nodule(s)	☐ Right upper lobe<br>☐ Right middle lobe<br>☐ Right lower lobe<br>☐ Left upper lobe<br>☐ Left lower lobe<br>((Register all affected lobes))
Placement of mass(es)	☐ Right upper lobe<br>☐ Right middle lobe<br>☐ Right lower lobe<br>☐ Left upper lobe<br>☐ Left lower lobe<br>((Register all affected lobes))

Describe consolidation(s) with other pattern	________________________
Placement of consolidation(s) with other pattern	☐ Right upper lobe<br>☐ Right middle lobe<br>☐ Right lower lobe<br>☐ Left upper lobe<br>☐ Left lower lobe<br>((Register all affected lobes))
Ground glass opacities (GGO) present	◯ Ja<br>◯ Nej
Placement of GGO	☐ Right upper lobe<br>☐ Right middle lobe<br>☐ Right lower lobe<br>☐ Left upper lobe<br>☐ Left lower lobe<br>((Register all affected lobes))
Cyst(s) present	◯ Ja<br>◯ Nej
Cysts pattern	◯ Localized<br>◯ Diffuse
Placement of cysts	◯ Right upper lobe<br>◯ Right middle lobe<br>◯ Right lower lobe<br>◯ Left upper lobe<br>◯ Left lower lobe<br>((Register all affected lobes))
Other Interstitial abnormalities?	◯ Ja<br>◯ Nej
Interstitial abnormality pattern	◯ Nodular<br>◯ Reticular<br>◯ Mixed<br>◯ Other
Describe other Interstitial abnormality pattern	______________________________
Honeycombing present?	◯ Ja<br>◯ Nej
Crazy-paving pattern present?	◯ Ja<br>◯ Nej
Mosaic perfusion present	◯ Ja<br>◯ Nej

Placement of mosaic perfusion	☐ Right upper lobe<br>☐ Right middle lobe<br>☐ Right lower lobe<br>☐ Left upper lobe<br>☐ Left lower lobe<br>((Register all affected lobes))
Tree-in-bud present	◯ Ja<br>◯ Nej
Placement of tree-in-bud	☐ Right upper lobe<br>☐ Right middle lobe<br>☐ Right lower lobe<br>☐ Left upper lobe<br>☐ Left lower lobe<br>((Register all affected lobes))
Bronchiectasis present	◯ Ja<br>◯ Nej<br>(((Excl. traction bronchiectasis)))
Type of bronchietasis	◯ Localized<br>◯ Diffuse
Placement of bronchiectasis	☐ Right upper lobe<br>☐ Right middle lobe<br>☐ Right lower lobe<br>☐ Left upper lobe<br>☐ Left lower lobe<br>((Register all affected lobes))
Emphysema present	◯ Ja<br>◯ Nej
Type of emphysema	◯ Centrilobular<br>◯ Panlobular<br>◯ Paraseptal<br>◯ Mixed
Placement of emphysema	☐ Right upper lobe<br>☐ Right middle lobe<br>☐ Right lower lobe<br>☐ Left upper lobe<br>☐ Left lower lobe<br>((Register all affected lobes))

Pleura cavities

Pneumothorax	◯ Ja<br>◯ Nej
Pneumothorax side	◯ Right<br>◯ Left<br>◯ Bilateral
Pneumothorax size	◯ Small<br>◯ Large<br>((Measurement according to BTS guidelines. In case)

Pleural effusion	◯ Ja<br>◯ Nej
Effusion configuration suspicious for possible empyema	◯ Ja<br>◯ Nej
Pleural effusion side	◯ Right<br>◯ Left<br>◯ Bilateral
Pleural effusion size	◯ Small<br>◯ Moderate<br>◯ Large<br>◯ Entire hemithorax<br>((In case of bilateral effusion, largest effusion)
Pleural thickening present	◯ Ja<br>◯ Nej
Pleural thickening side	◯ Right<br>◯ Left<br>◯ Bilateral

Other findings

Bone pathology	◯ Ja<br>◯ Nej
Describe bone pathology	__________________________________
Other findings	◯ Ja<br>◯ Nej
Describe other findings	____

Conclusion and confidence

CT Image quality	◯ 1) Poor image quality: it is not possible to recognise any anatomical structures<br>◯ 2) Some anatomical structures can be visualised, but it is still not possible to diagnose or exclude any pathology<br>◯ 3) Suboptimal image quality: Some anatomical structures can be visualised, and it is possible to diagnose or exclude rough pathology<br>◯ 4) All relevant anatomical structures and any potential pathology can be visualised, but still the resolution of the image are not perfect.<br>◯ 5) All relevant anatomical structures and any potential pathology can be visualised and the resolution of the picture are near perfect
Is pneumonia the most likely diagnosis?	◯ Ja<br>◯ Nej

Suspected diagnosis based on CT	☐ Normal findings<br>☐ Pneumonia<br>☐ Non-cardiogenic pulmonary oedema<br>☐ Cardiogenic pulmonary oedema<br>☐ Pneumonia with parapneumoic effusion<br>☐ Empyema<br>☐ Pleural effusion of unknown origin<br>☐ Pulmonary embolism<br>☐ Pneumothorax<br>☐ COPD<br>☐ Asthma<br>☐ Interstitial lung disease<br>☐ Malignancy<br>☐ Other<br>((Several diagnoses may be chosen))
Describe other suspected diagnosis	________________________________<br>((Several diagnoses may be chosen))
Diagnostic confidence in CT findings	0 50 100<br>*(Place a mark on the scale above)*
Based on HRCT findings, is additional imaging needed?	◯ Ja ◯ Nej
Additional imaging needed	☐ Erect CXR in 2 planes<br>☐ CT of the chest ☐ HRCT of the chest<br>☐ MR of the chest ☐ PET-CT<br>☐ Echocardiography ☐ Lung ultrasound<br>☐ Ventilation / perfusion scintigrafi<br>☐ Other
Which other type of additional imaging is needed?	________________________________
Time when CT assessment ends	________________________________
