# Supplementary material for: Handheld Ultrasound Devices Used by Newly Certified Operators for Pneumonia in the Emergency Department—A Diagnostic Accuracy Study
Source: Diagnostics (Basel). 2024 Aug 30;14(17):1921. doi: 10.3390/diagnostics14171921 (PMC11394211; doi:10.3390/diagnostics14171921)
Supplement: Supplementary file 1 [file diagnostics-14-01921-s001.zip › File S4 - FLUS positive for pneumonia negative ekspert diagnoser.pdf]

File S4 - FLUS positive for pneumonia negative expert diagnoses

FLUS positive for pneumonia<br>Expert diagnoses: No pneumonia<br>n=20 (24%)	
No infection	11
Acute upper respiratory infections	2
UTI with systemic symptoms	2
Endocarditis	2
Abscess of lung and mediastinum	1
Pyogenic artritis	1
UTI with systemic symptoms	1

FLUS: focused lung ultrasound; UTI: urinary tract infection
